# Endothelial activation, haemostasis and thrombosis biomarkers in Ugandan children with severe malaria participating in a clinical trial

**DOI:** 10.1186/s12936-016-1106-z

**Published:** 2016-02-02

**Authors:** Susan M. Graham, Junmei Chen, Dominic W. Chung, Kevin R. Barker, Andrea L. Conroy, Michael T. Hawkes, Sophie Namasopo, Kevin C. Kain, José A. López, W. Conrad Liles

**Affiliations:** Department of Medicine, University of Washington, Box 359909, 325 Ninth Avenue, Seattle, WA 98104 USA; Department of Global Health, University of Washington, Seattle, WA USA; Department of Epidemiology, University of Washington, Seattle, WA USA; Bloodworks Research Institute, Seattle, WA USA; Department of Laboratory Medicine and Pathobiology, University of Toronto, Toronto, ON Canada; University Health Network-Toronto General Hospital, Toronto, ON Canada; Sandra Rotman Centre for Global Health, Toronto, ON Canada; Department of Medicine, Indiana University, Indianapolis, IN USA; Department of Pediatrics, University of Alberta, Edmonton, AB Canada; Jinja Regional Referral Hospital, Jinja, Uganda; Department of Biochemistry, University of Washington, Seattle, WA USA; Department of Pathology, University of Washington, Seattle, WA USA

**Keywords:** Malaria, Endothelial activation, Von Willebrand factor, Angiopoietins, Syndecan-1

## Abstract

**Background:**

Malaria is a major cause of morbidity and mortality in sub-Saharan Africa, and poor outcomes have been associated with endothelial activation. In this study, biomarkers of endothelial activation, haemostasis, and thrombosis were measured in Ugandan children with severe malaria who participated in a clinical trial, in order to investigate associations between these processes.

**Methods:**

Serum and plasma were collected from participants at baseline (day 1), and on days 2, 3, 4, and 14. Von Willebrand factor (VWF) antigen was measured in stored plasma samples from all trial participants, and its association with mortality and changes over time were analysed. VWF multimer patterns were evaluated in baseline serum samples by gel electrophoresis followed by Western blotting. Levels of angiopoietins 1 and 2, VWF antigen, total active VWF, ADAMTS13, platelet counts, apolipoprotein A1, and syndecan-1 were measured in stored serum samples from 12 survivors at baseline and day 4.

**Results:**

VWF antigen levels were associated with mortality, and decreased over time in survivors. Baseline VWF antigen and total active VWF levels were elevated, and very large multimers were present in the baseline serum of several patients. Higher platelet counts were associated with higher angiopoietin-1 and apolipoprotein A1 levels, while lower platelet counts were associated with higher syndecan-1, a marker of endothelial damage. Higher angiopoietin-2 to angiopoietin-1 ratio and higher syndecan-1 levels were correlated with lower apolipoprotein A1 levels. There were no correlations between total active VWF, VWF antigen, or ADAMTS13 levels and the other biomarkers at baseline. Changes in biomarker levels between baseline and day 4 were not correlated.

**Conclusions:**

These results confirm that severe malaria is associated with endothelial activation, and suggest that endothelial activation contributes to microvascular thrombosis and endothelial damage.

**Electronic supplementary material:**

The online version of this article (doi:10.1186/s12936-016-1106-z) contains supplementary material, which is available to authorized users.

## Background

Malaria is a major cause of morbidity and mortality in sub-Saharan Africa, where over 90 % of all malaria deaths occur [1]. Children are most affected, with approximately 78 % of malaria deaths worldwide occurring in children under 5 years of age [1]. While most infections can be managed effectively if promptly diagnosed and treated, some patients present with severe forms of malaria, including cerebral malaria (CM), or worsen despite treatment. Fatality rates for children with severe malaria (SM) and CM have been estimated at approximately 8.5 and 18 % despite treatment with intravenous artesunate, the current standard of care [2]. Survivors of CM can develop neurocognitive deficits, including epilepsy, behavioural disorders, and motor, sensory or language deficits [3]. These outcomes suggest that adjunctive therapy targeting mechanistic pathways mediating severe disease is needed.

Evidence suggests that the host response plays an important role in determining disease severity and outcomes [4, 5]. Systemic inflammation in SM is associated with endothelial activation and expression of adhesion molecules that can amplify inflammatory signals [6]. Endothelial activation involves dysregulation of the angiopoietin/receptor tyrosine kinase Tie2 (Ang/Tie2) axis [7], as well as the release of Von Willebrand Factor (VWF) from secretory granules known as Weibel–Palade bodies. VWF is an important multimeric protein necessary for normal haemostasis; when dysregulated, VWF can cause devastating microvascular thrombosis [8]. Elevated VWF levels have been reported in children with asymptomatic parasitaemia [9], suggesting that malaria parasites induce a state of endothelial activation even at early stages of infection.

VWF is normally cleaved from the surface of endothelial cells by the activity of ADAMTS13, a zinc-containing metalloprotease [10]. In malaria, however, ADAMTS13 activity is reduced and VWF can persist and self-associate into large multimers which can bind platelets [8]. These bound platelets can accumulate on the endothelial surface, causing thrombi and leading to vessel occlusion [11]. Systemic endothelial activation in SM is also accompanied by a reduced plasma or serum concentration of apolipoprotein A1 (ApoA1), the primary protein component of high-density lipoprotein (HDL) particles [12]. Published work by the authors of this paper has demonstrated that plasma HDL and ApoA1 dose-dependently decrease the extent of VWF self-association, in the process also significantly decreasing the binding of platelets to the vessel wall [13]. ApoA1 levels may, therefore, be protective in SM. Conversely, syndecan-1, a transmembrane heparin sulfate proteoglycan involved in cell signalling, is associated with coagulopathy in severe sepsis [14]. Higher levels of syndecan-1 in plasma or serum indicate damage to the glycocalyx on the apical surface of endothelial cells [15].

Endothelial activation, mediated by dysregulation of the Ang/Tie2 axis and acute release of VWF, may produce a systemic microangiopathy in SM, contributing to morbidity and mortality. In this study, biomarkers of endothelial activation, haemostasis and thrombosis, including angiopoietin 1 and 2 (Ang-1 and Ang-2) levels, VWF antigen, total active VWF, and ADAMTS13, platelet counts, syndecan-1 levels, and ApoA1 levels, were measured among Ugandan children with SM who participated in a clinical trial [16], in order to investigate associations between these processes.

## Methods

### Ethics and consents

Ethical approval was granted by the Makerere University School of Medicine Research Ethics Committee (Protocol #2010-107), the Uganda National Council for Science and Technology (Ref: HS857), the National Drug Authority of Uganda (Ref: 297/ESR/NDA/DID-01/2011), and the University Health Network Research Ethics Committee, Toronto, Canada (UHN REB#10-0607-B). Written informed consent was provided by an accompanying parent or caregiver for all study participants. The clinical trial from which participant samples derived was registered (Clinical trials.gov identifier: NCT01255215).

### Patients and sample

One-hundred and eighty Ugandan children between the ages of 1 and 10 years who were admitted to the Jinja Regional Referral Hospital with SM were enrolled into a randomized controlled trial of adjunctive therapy with inhaled nitric oxide [16, 17]. Malaria was diagnosed by light microscopy of Giemsa-stained thick smears of EDTA-anticoagulated peripheral blood and by three-band rapid diagnostic tests with *Plasmodium falciparum* histidine rich protein 2 (HRP2) and pan-malaria lactate dehydrogenase (pLDH) (First Response Malaria Ag. HRP2/pLDH Combo Rapid Diagnostic Test, Premier Medical Corp Ltd, India) [18].

SM was defined as malaria in patients experiencing repeated seizures (i.e., two or more generalized seizures in 24 h), prostration, impaired consciousness (i.e., Blantyre coma score <5), or respiratory distress (i.e., age-relative tachypnea with sustained nasal flaring, deep breathing or sub-costal retractions) [16]. Children were excluded from the trial if they had received quinine prior to enrolment, could not tolerate a mask for gas delivery, were suspected to have acute bacterial meningitis, or had methaemoglobin >2 % at baseline, known chronic illness (e.g., renal, cardiac, hepatitis disease, diabetes, epilepsy, cerebral palsy, haemoglobinopathy, or AIDS), or severe malnutrition (i.e., symmetrical oedema involving at least the feet or weight-for-length or height more than three standard deviations below the mean based on WHO reference charts). All children received intravenous artesunate. There was no effect of inhaled nitric oxide on treatment outcomes [16].

Blood was collected daily in plastic K2EDTA BD Microtainer^®^ tubes (New Jersey, USA) according to standard operating procedures. Briefly, 500 μL of blood was added to the microtainer tubes, which were then inverted ten times, and centrifuged for 20 min at 4500 rpm before plasma was collected, taking care not to disturb the buffy layer, and transferred to cryovials for storage. Serum samples were collected in red-top tubes, in which blood was allowed to clot for 30–60 min before centrifugation for 20 min at 4500 rpm, followed by transfer to cryovials for storage. Both plasma and serum were kept up to a maximum of 24 h at 4 °C before storing at −80 °C until use. Both sample types were collected at baseline day 1, day 2, day 3, day 4, and at day 14.

### Laboratory testing, all participants

Baseline complete blood counts, including platelet counts, were obtained at a regional reference laboratory using a Beckman Coulter AcT 5 Diff hematology analyzer (Beckman Coulter, Inc, Fullerton, CA, USA). For the evaluation of change in VWF antigen over time, VWF antigen was measured in stored plasma samples from all study participants at all available time-points (i.e., through day 14 or death). Plates were coated with anti-human VWF antibody (DAKO North America, Inc, Carpinteria, CA, USA, 1:600 dilution), incubated with samples (1:1000 dilution) and standards (serial dilutions of recombinant VWF, American Diagnostica, Stamford, CT, USA), then incubated with HRP-conjugated anti-human VWF (DAKO North America, Inc, Carpinteria, CA, USA, 1:8000 dilution). Bound antibodies were detected with the HRP substrate tetramethylbenzidine, the reaction was stopped with H_2_SO_4_, then the colour signal was read at 450 nm. Background signal was determined from blank wells included on each plate (assay buffer added instead of sample), and background optical density was subtracted from all samples and standards prior to analysis. Samples with optical densities below the lowest detectable standard were assigned the value of that standard.

### Laboratory testing, detailed subset

Baseline and day 4 serum samples from a sub-set of 12 patients who survived until day 14 and had sample available were shipped to the López laboratory in Seattle. VWF multimer patterns were evaluated in the baseline serum samples of this participant subset and in pooled normal plasma by 1 % agarose gel electrophoresis followed by Western blotting with a polyclonal VWF antibody, as described previously [19], and densitometry was quantified by ImageQuant TL software (GE Healthcare Bio-Sciences, Pittsburgh, PA, USA). For baseline and day 4 serum samples from this participant sub-set, VWF antigen levels were determined by ELISA using a polyclonal VWF antibody (DAKO North America, Inc, Carpinteria, CA, USA) as the capture antibody, with detection of bound VWF by an HRP-conjugated VWF antibody (DAKO North America, Inc, Carpinteria, CA, USA). VWF activation factor was determined by ELISA using llama nanobody AU/VWFa-11 as the capture antibody [20]. Total active VWF, a measure of total VWF reactivity, was calculated by multiplying VWF antigen (expressed as fold of the control sample) by VWF activation factor, as described by Chen et al. [21]. Ang-1, Ang-2, ADAMTS13, and syndecan-1 levels were measured using commercially available ELISA kits (R&D Systems, Minneapolis, MN, USA) according to manufacturer’s instructions. The ApoA1 antigen level was measured by ELISA using a monoclonal ApoA1 antibody as the capture antibody and a horseradish peroxidase (HRP)-conjugated polyclonal ApoA1 antibody as the detection antibody (LS-C11247 and LS-C11248, respectively, LifeSpan Biosciences, Inc, Seattle, WA, USA). Participant biomarker data for VWF antigen, total active VWF, ADAMTS13, ApoA1, and syndecan-1 were compared to data from plasma pooled from 20 normal donors (Cryocheck Normal Reference Plasma, Precision Biologic, Dartmouth, NS, Canada) to determine fold-normal values for this analysis. Measurements of VWF antigen in serum and plasma are closely correlated (Chen, unpublished observations).

### Data analysis

All biomarker data were first tested for normality using the Shapiro–Wilk test, and log_10_-transformed to approximate normality if not normally distributed. Change in VWF antigen over time was evaluated in all 179 participants for whom data were available after baseline, using overlaid longitudinal plots of VWF antigen by day of hospitalization. Generalized estimating equations (GEE) with a logit link and exchangeable correlation matrix were used to evaluate the association of VWF antigen levels with death. GEE with an identity link and exchangeable correlation matrix were used to evaluate change in VWF antigen levels by hospital day, overall and in survivors.

In the sub-set with detailed biomarker data, t tests were used to compare VWF antigen levels and total active VWF levels to the normal value expected (i.e., one-fold-normal). Pair-wise correlations were calculated between all measured biomarkers at the pre-treatment baseline, and scatter plots with fitted regression lines were created. Changes in each biomarker (except for platelets, which were not measured after baseline) between the pre-treatment baseline and day 4 were calculated, and pair-wise correlations were also calculated between these measures. Stata version 12.1 was used for analysis of data (Additional files 1, 2).

## Results

### Results, all participants

At baseline, the geometric mean parasite density was 17,399 (range, 0–696,320) parasites per μL. Of note, in ten cases, no parasites were seen on the admission blood smear, despite a positive-screening rapid diagnostic test result. In one other case, the species was diagnosed as *Plasmodium ovale*. In all of these cases, polymerase chain reaction testing performed on the red blood cell pellet was positive for *P. falciparum.* The mean platelet count was 106 (range, 2–489) × 10^9^ per L, and mean VWF antigen level was 93.3 (range, 14.8–322.7) ng/mL. While parasite density was negatively correlated with platelet count (r = −0.234, p = 0.002) and positively correlated with VWF antigen (r = 0.178, p = 0.018), the correlation between platelet count and VWF antigen was not significant (r = 0.095, p = 0.224).

**Fig. 1:**
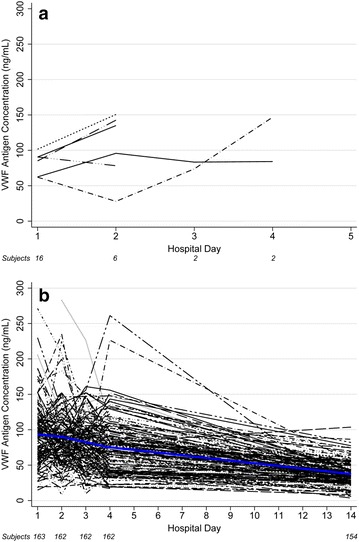
Change in VWF antigen over time during hospitalization. Overlaid longitudinal plots were used to represent the change in VWF antigen levels over time in non-survivors (**a**) and survivors (**b**). Each line represents the data points for one participant. The *blue line* in **b** is a Loess estimation of the rate of decrease over time. Numbers on the *X axis* detail the number of subjects with data at each time point

Sixteen participants died during hospitalization, with 14 of these deaths (87.5 %) occurring within 48 h of admission. Among the 179 participants for whom VWF antigen measures were available at baseline and during follow-up, there was a 3.39-fold (95 % confidence interval (CI), 1.08–10.62, p = 0.036) increase in the odds of mortality for each unit increase in log_10_-transformed VWF antigen level. VWF antigen levels decreased by 0.031 log_10_ ng/mL (95 % CI, 0.034–0.028 log_10_ ng/mL) per day overall and in the 163 survivors (p < 0.001 for both analyses). Adjustment for treatment group (i.e., inhaled nitric oxide or control) did not change these results. VWF antigen data were available for the children who died at baseline for ten, day 2 for six, days 3 and 4 for two, and day 14 for none. In several of these cases, VWF antigen levels actually increased over time before death. Figure 1 presents the change in VWF antigen levels over time in non-survivors (panel A) and survivors (panel B).

### Results, detailed sub-set

**Fig. 2:**
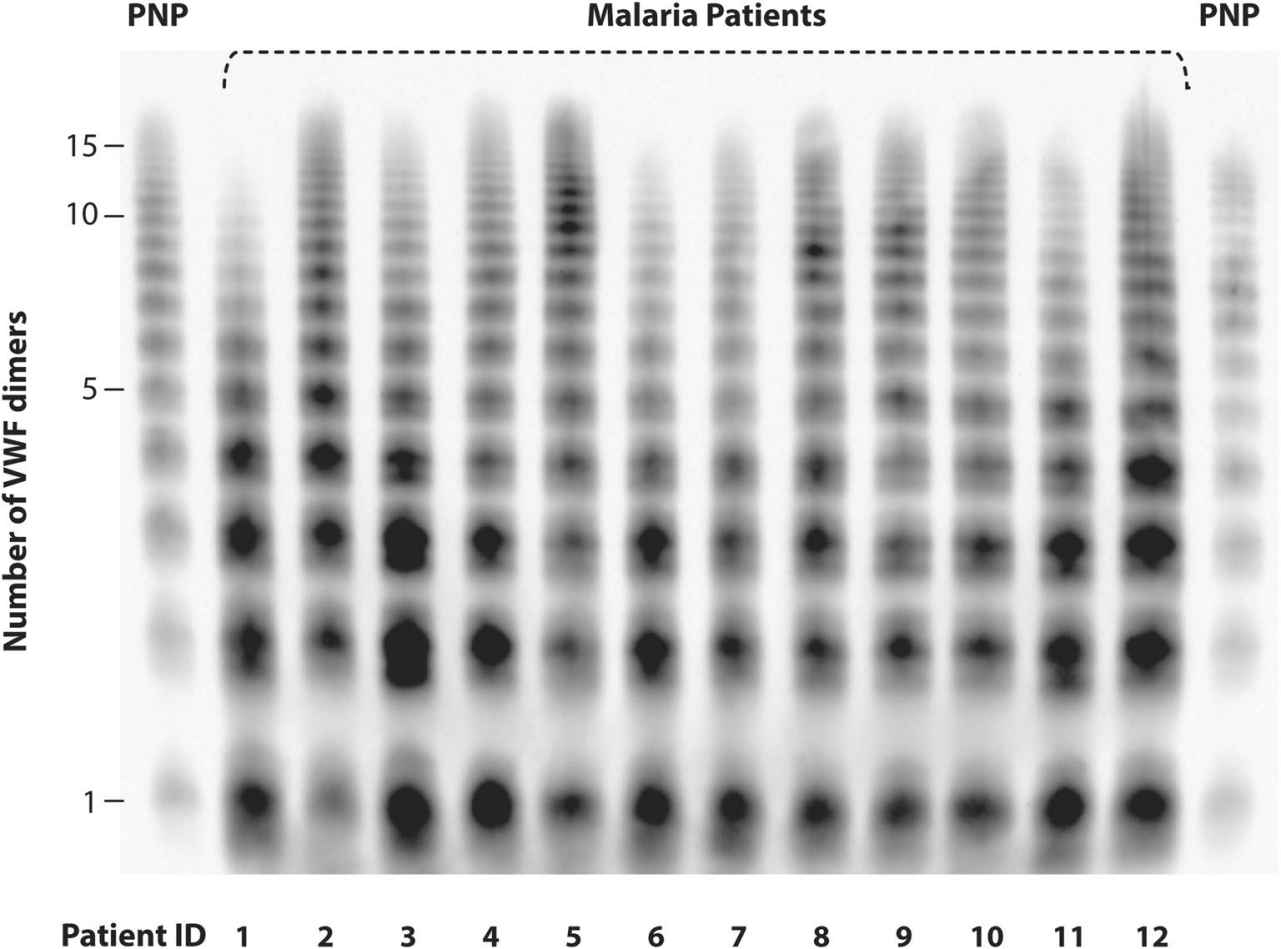
VWF multimer distribution. The VWF multimer distribution was examined in baseline serum samples from either SM patients or pooled normal plasma (PNP) by electrophoresis on a non-reducing 1.7 % agarose-SDS gel, followed by western blotting. The *Y axis* shows the approximate number of VWF dimers at each level of the gel. Patients 2, 4, 5, 8–10, and 12 had larger VWF multimers than those found in normal plasma, with two to four larger bands (on top of the multimers) detected by densitometry analysis using ImageQuant TL software (GE Healthcare Bio-Sciences, Pittsburgh, PA, USA)

Among the 12 participants with more detailed assessments, baseline VWF levels (median 3.52-fold normal, range 1.72–4.67-fold normal, p < 0.0001) and total active VWF (median 6.25-fold normal, range 3.01–19.19-fold normal, p = 0.0002) were both significantly elevated compared to normal. By agarose gel electrophoresis, several patients had larger VWF multimers than those found in normal plasma, with two to four bands larger than the largest detected in pooled normal plasma by densitometry analysis (Fig. 2).

Table 1 presents the absolute values of and correlations between the biomarkers studied in baseline serum samples. While higher platelet counts were associated with higher log_10_- transformed Ang-1 (r = 0.62, p = 0.04) and ApoA1 levels (r = 0.61, p = 0.05), platelet count was inversely associated with syndecan-1 levels (r = −0.68, p = 0.02). ApoA1 levels were inversely correlated with the log_10_-transformed Ang-2/Ang-1 ratio (r = −0.63, p = 0.03) and with syndecan-1 levels (r = −0.68, p = 0.01). As expected, Ang-2 and log_10_-transformed Ang-1 levels were both correlated with the log_10_-transformed Ang-2/Ang-1 ratio.

**Table 1:** Correlations between biomarkers at baseline in 12 children with severe malaria

Biomarker (units)	Mean (range)	Platelets	Log_10_Ang-1	Ang-2	Log_10_ Ang-2 to Ang-1 ratio	Syndecan-1	ApoA1	VWF antigen	Total active VWF
Platelets (× 10^9^ per L)	92 (16–255)								
Log_10_ Ang-1 (log_10_ng/mL)	0.35 (−1.10–1.29)	*r* = *0.6248 p* = *0.0399*							
Ang-2 (ng/mL)	13.97 (3.86–26.71)	r = −0.1494 p = 0.6612	r = −0.4730 p = 0.1204						
Log_10_Ang-2 to Ang-1 ratio	0.73 (−0.70 to 2.52)	r = −0.5533 p = 0.0775	*r* = −*0.9592 p* < *0.0001*	*r* = *0.6945 p* = *0.0122*					
Syndecan-1 (fold-normal)	7.41 (3.44–11.12)	*r* = −*0.6790 p* = *0.0216*	r = −0.4881 p = 0.1074	r = 0.4358 p = 0.1568	r = 0.5226 p = 0.0813				
ApoA1 (fold-normal)	0.86 (0.40–1.45)	*r* = *0.6051 p* = *0.0486*	r = 0.5687 p = 0.0537	r = −0.4707 p = 0.1225	*r* = −*0.6322 p* = *0.0274*	*r* = −*0.6814 p* = *0.0147*			
VWF Antigen (fold-normal)	3.39 (1.72–4.67)	r = 0.0310 p = 0.9280	r = −0.1336 p = 0.6789	r = 0.5071 p = 0.0924	r = 0.2691 p = 0.3977	r = 0.0942 p = 0.7709	r = −0.1729 p = 0.5911		
Total active VWF (fold-normal)	8.27 (3.01–19.19)	r = −0.0091 p = 0.9787	r = −0.0631 p = 0.8455	r = −0.4849 p = 0.1100	r = −0.1118 p = 0.7293	r = −0.2046 p = 0.5236	r = 0.1813 p = 0.5729	r = 0.0868 p = 0.7885	
ADAMTS13 (fold-normal)	0.90 (0.58–1.27)	r = −0.0328 p = 0.9237	r = 0.0064 p = 0.9843	r = −0.0394 p = 0.9033	r = −0.0001 p = 0.9999	r = −0.2614 p = 0.4119	r = 0.3197 p = 0.3111	r = 0.3833 p = 0.2187	r = 0.2844 p = 0.3702

NB Significant correlations are indicated in italics

**Fig. 3:**
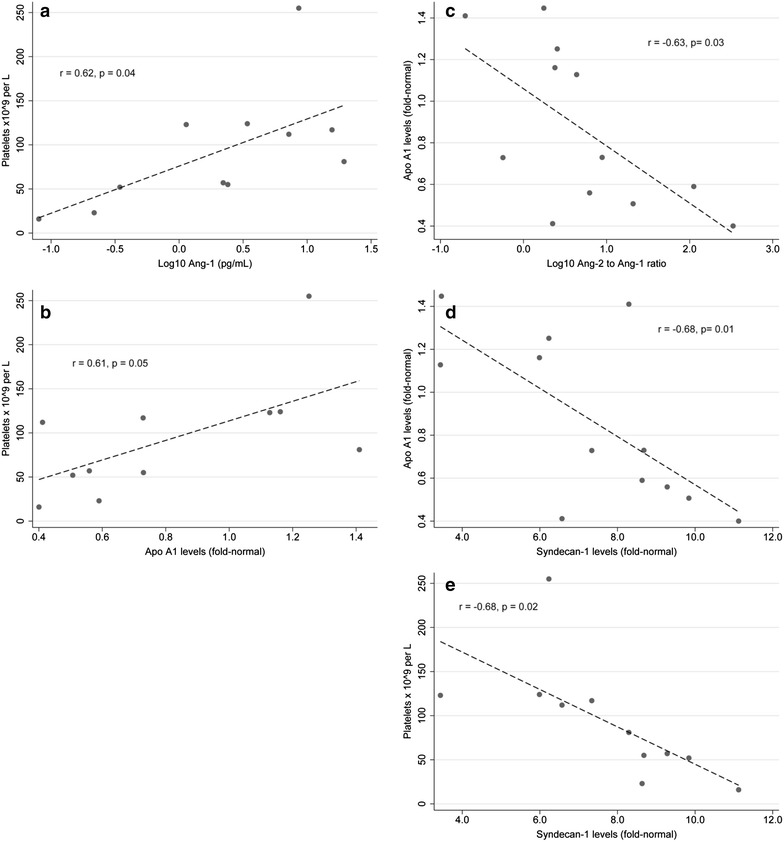
Scatterplots with fitted regression lines. Data points are represented by individual dots, and the dashed line represents the fitted regression. The Pearson correlation and corresponding p value are also included. Panels present **a** platelet counts and log_10_-transformed Ang-1; **b** platelet counts and ApoA1 levels; **c** ApoA1 levels and log_10_-transformed Ang-2 to Ang-1 ratio; **d** ApoA1 levels and syndecan-1 levels; and **e** platelet counts and syndecan-1 levels

Figure 3 presents scatterplots with fitted regression lines for the significant correlations identified at the pre-treatment baseline. There were no correlations of platelets, Ang-2 or Ang-1 levels or ratio, syndecan-1 levels, or ApoA1 levels with total active VWF, VWF antigen levels, or ADAMTS13 levels. In addition, there were no significant correlations between biomarkers with respect to change from the pre-treatment baseline to day 4 after treatment.

## Discussion

In this study of Ugandan children with SM, higher VWF antigen levels were associated with an increased risk of mortality, and VWF antigen levels decreased in survivors but not in non-survivors for the limited time they were followed. In the sub-set of patients for whom detailed measurements were performed, platelet counts were positively correlated with log_10_-transformed Ang-1 and with ApoA1 levels (both protective factors), while platelet counts were negatively correlated with syndecan-1 levels (indicative of endothelial glycocalyx damage). Higher log_10_-transformed Ang-2/Ang-1 ratio and higher syndecan-1 levels, both indicative of endothelial activation and inflammation, were associated with lower ApoA1 levels. Although none of these measures correlated with VWF antigen, total active VWF, or ADAMTS13 levels, the SM patients in this study had elevated VWF levels and activity, with ultra-large multimers present in the serum of several patients. These findings reflect a state of VWF persistence and self-association, which increases platelet binding and the risks of thrombosis and microvascular occlusion.

In multiple field studies, this and other research groups have shown that altered levels of the endothelial regulators Ang-1 and Ang-2, as well as other markers of endothelial activation (e.g., soluble intercellular adhesion molecule-1, VWF antigen), are associated with malarial disease severity and mortality in both adults and children [22–27]. In these studies, Ang-2 was an independent and quantitative marker of malaria-related morbidity and mortality and a better predictor of outcome than other measures, including lactate. In a study of Malawian children, Ang-1 and Ang-2 levels on admission were highly predictive of malarial retinopathy and CM [24]. Moreover, they were superior to retinopathy in predicting mortality in children presenting with WHO-defined CM [24]. While a correlation between Ang-1 and platelet counts might be expected in serum due to release of platelet-stored Ang-1 upon clot formation [28], both Ang-1 and Ang-2 levels in serum have been prognostically informative [22].

Very high plasma or serum concentrations of VWF, indicative of marked endothelial activation, have been previously described in SM caused by *P. falciparum* infection [23, 29, 30]. In addition, VWF antigen levels have been reported to decrease with treatment [31]. A rapid rise in VWF such as was seen in some non-survivors in this study, has been associated with a fall in the platelet count, a process that commences early after the onset of blood-stage infection [29]. As in the present study, VWF antigen in plasma or serum during SM has been reported to be enriched in ultra-large and hyperadhesive forms [29, 32]. While no associations were identified between VWF antigen, total active VWF, and ADAMTS13 and other biomarkers examined in the current study, in vitro studies have demonstrated that the ability of VWF to support platelet adhesion on intact endothelium is tightly controlled by competing processes: specifically, the rates of VWF self-association and platelet binding *versus* the rate of ADAMTS13 cleavage [11, 13]. While little is known about the role of VWF self-association in malaria, both VWF and platelets likely play an important pathophysiological role in SM, functioning to facilitate the attachment of infected erythrocytes and directly occluding the vessel. The role of glycocalyx damage in this process also merits further investigation, given the finding in this study of the inverse correlation between platelet counts and syndecan-1 levels.

In ongoing in vitro studies, this research group has found that ApoA1 can potently inhibit VWF self-association and consequent platelet accumulation [13], and has also shown that in sepsis, ApoA1 levels correlate inversely with total active VWF [13]. Total active VWF is a parameter that takes into account both VWF antigen concentration and adhesive activity, as determined by the binding of a llama nanobody that recognizes an epitope exposed in the VWF A1 domain when VWF binds to platelets [20]. Low ApoA1 levels could exacerbate the course of malarial infection in at least two ways. First, VWF strands will be more likely to accumulate on vessel walls and become resistant to proteolytic removal because of unabated self-association [13]. Second, ApoA1 and HDL particles are necessary to protect prostacyclin, a potent platelet inhibitor, from rapid degradation, increasing the reactivity of platelets [33]. In this study, higher ApoA1 levels were associated with higher Ang-1 levels, and lower ApoA1 levels were associated with a higher Ang-2/Ang-1 ratio. Lower ApoA1 levels were also correlated with lower platelet counts and higher syndecan-1 levels, suggesting that lower ApoA1 was associated with microvascular thrombosis and endothelial damage.

Several limitations of this study warrant discussion. First, the study was conducted in the context of a randomized clinical trial of inhaled nitric oxide as an adjunctive treatment [16]. Clinical outcomes, including circulating angiopoietin 2 levels, did not differ by study arm [16], and adjustment for treatment group did not affect the analysis of change in VWF antigen over time. In addition, the detailed sub-set analysis focused on data from samples collected prior to the administration of nitric oxide or room air placebo. Second, no platelet counts were available after baseline, so it was not possible to evaluate how changes in VWF antigen over time or how detailed measures in the sub-set related to changes in platelet counts. Third, due to limited sample volume and the cost of additional assays, the sub-set in which detailed analyses were performed was limited. Nevertheless, this study has detected significant correlations that highlight the importance of endothelial activation in SM.

## Conclusion

These results confirm that SM is associated with endothelial activation and suggest that higher levels of endothelial activation are associated with microvascular thrombosis and endothelial damage. These findings may help elucidate pathogenic mechanisms of endothelial activation and microangiopathy in SM that could lead to the identification of new prognostic biomarkers and the development of therapeutic interventions. Further research to investigate causality and potential interventions for these implicated pathways could address critical knowledge gaps in the understanding of the pathobiology of SM and facilitate the identification of clinically informative prognostic biomarkers, thereby enabling risk stratification and targeting of interventions.

## Availability of supporting data

The data sets supporting the results of this article are available as additional files.

## Additional files

10.1186/s12936-016-1106-z Longitudinal dataset. This dataset was used for analysis of change in VWF antigen over time. The file is available as an excel spreadsheet.
10.1186/s12936-016-1106-z Biomarkers for detailed sub-set analysis. This dataset was used for analysis of correlations of biomarkers at baseline and change in biomarkers from baseline to day 4. The file is available as an excel spreadsheet.
